# Anatomical and functional outcomes after hysterectomy and bilateral sacrospinous ligament fixation for stage IV uterovaginal prolapse: a prospective case series

**DOI:** 10.1186/s12894-020-00694-3

**Published:** 2020-08-19

**Authors:** Tilemachos Kavvadias, Birgitt Schoenfisch, Sara Yvonne Brucker, Christl Reisenauer

**Affiliations:** 1 Department of Obstetrics and Gynecology, University Hospital Tübingen, Calwerstrasse 7, 72076 Tübingen, Germany; 2 Department of Gynecology and Obstetrics, University Hospital Basel, Spitalstrasse 21, 4031 Basel, Switzerland

**Keywords:** Native tissue repair; Pelvic organ prolapse; Bilateral sacrospinous ligament fixation

## Abstract

**Background:**

Aim of this study is to examine pelvic floor symptoms, anatomical results and patients’ satisfaction after sacrospinous vaginal fixation for stage 4 pelvic organ prolapse.

**Methods:**

All patients with stage 4 pelvic organ prolapse were treated with vaginal hysterectomy, native tissue cystocele and rectocele repair and bilateral sacrospinous vaginal fixation. Anatomical and functional outcomes according to the POPq classification system and the German version of the Australian pelvic floor questionnaire were assessed. Changes between baseline, first follow-up and second follow-up were assessed by the paired Wilcoxon rank test using R, version 3.5.1.

**Results:**

20 patients were included in the study. Scores in all four domains of the pelvic floor symptom questionnaire (bladder, bowel, prolapse, sexual function) were significantly improved at 6 and 12-months follow-up. One patient presented with a symptomatic stage 3 cystocele that needed a second surgical intervention and two patients needed surgery due to a de novo stress urinary incontinence. There were no perioperative adverse events and all patients reported full satisfaction after surgery.

**Conclusions:**

The vaginal approach with hysterectomy, native tissue repair and bilateral sacrospinous vaginal fixation seems to be a safe and effective method for the treatment of advanced stage POP, offering excellent relief in all pelvic floor symptoms.

**Trial registration:**

ClinicalTrials.gov (NCT 02998216), December 20th, 2016.

Prospectively registered.

## Background

Pelvic organ prolapse (POP) is defined as a ‘downward displacement’ of the uterus and /or the vaginal compartments and their neighbouring organs such as bladder, rectum and bowel, causing bothersome symptoms and affecting a woman’s quality of life [1]. It has been reported that in a general population 40% of women aged between 45 and 85 years have an objective POP on examination, but only 12% of these women are symptomatic [2]. Conservative treatment with a pessary is often considered as first line therapy, with surgery reserved for patients who decline or fail management of POP with a pessary. While the choice of procedure depends on severity of POP, patients’ goals and surgeons’ expertise, vaginal native tissue repair without using synthetic mesh or graft materials is considered as appropriate surgical treatment option for most women with primary POP [3, 4]. Furthermore, it is very common that surgical treatments for anterior, posterior and apical pelvic floor support loss are combined, in order to achieve optimal results [4, 5].

Apical support is of great importance for the stability of the pelvic floor. There is a growing understanding that adequate vaginal apex support is essential for a durable surgical repair in women with advanced prolapse [6]. Moreover, surgical correction of the anterior and posterior wall may fail unless the apex is adequately supported [7]. Vaginal sacrospinous ligament fixation (SSLF) is one of the most common procedures performed for the restoration of apical support loss and is considered effective and safe. It shows a subjective success rate of 84–99% and an objective success rate of 67–93% [8]. SSLF can be performed unilaterally or bilaterally and it has the advantage that no synthetic material or graft is needed. The bilateral approach however has been criticized for insufficient midline support, leaving the central part of the apex without support and vulnerable to intra-pelvic pressure on the genital hiatus, although this is more of an objective outcome and may not affect subjective outcomes [8]. Furthermore, additional midline support may be provided by concomitant anterior and posterior repair, but the existing evidence is sparse, mainly observational, and retrospective. It is only limited to anatomical results and only few of the included patient in previous studies on the efficacy of SSLF had advanced POP (stage IV) [6].

The aim of this pilot study is to prospectively evaluate the efficacy of vaginal bilateral sacrospinous ligament fixation for POPQ stage IV and to examine the impact on patients’ symptoms at six and twelve months follow-up after surgery.

## Methods

The study protocol was approved by the Ethics Committee at the University Hospital of Tübingen (Ethikkommission Universitätskliikum Tübingen) (ref. number: 130/2017BO2) and was registered with ClinicalTrials.gov (NCT 02998216).

**Fig. 1:**
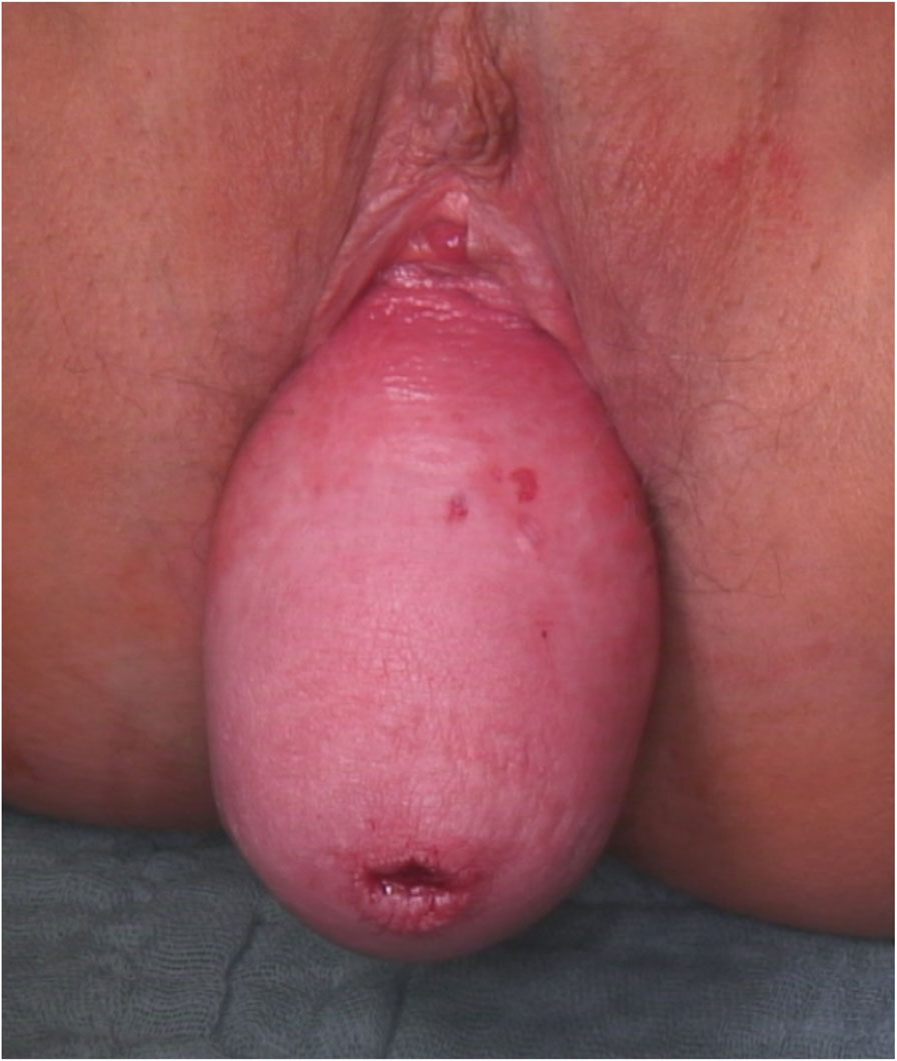
Pelvic organ prolapse stage IV according to POP-Q system

This prospective interventional pilot, single centre, non-randomized study was conducted at the Department of Obstetrics and Gynecology Tübingen in the period from March 2017 to March 2018. All women suffering from symptomatic uterovaginal POP stage IV (Fig. 1), according to the Pelvic Organ Prolapse Quantification (POP-Q) system [1], were included in the study.

Exclusion criteria were previous POP or incontinence surgeries, age < 18 and > 75 years, participation in other clinical trials, active malignant disease and inability to understand the purpose of the study and sign the informed consent. The exclusion of women over 75 was made on the basis of recruiting a homogenous group of women who would profit from the procedure and would not require or prefer an obliterative procedure for the correction of their prolapse. We also had to provide an upper cut-off age in our inclusion criteria for anesthesiological reasons, to avoid including fragile and multi-morbid patients.

The primary outcome measure was the improvement of the pelvic floor symptoms and the secondary outcome measure was the anatomical correction of prolapse.

Pelvic floor symptoms were assessed using the self-administered, validated German version of the Australian female pelvic floor questionnaire. Questions are grouped according to the physiological functions of the pelvic floor, i.e. bladder function, bowel function, prolapse symptoms, and sexual function domains. The scores are then calculated on a 0 to 10 scale on each domain, ranging from less to most severe and giving a potential final overall score of maximum 40 [9, 10]. Patients were asked to answer the female pelvic floor questionnaire before, as well as 6 and 12 months after surgery. Additionally, a complete staging using the POP-Q system was performed using a standard measurement tape and patients’ health condition was measured on 5-point Likert scales (1 = much worse, 5 = much better). The POP-Q quantification was not performed by the surgeon, in order to avoid bias. Epidemiological data, such as age, parity, comorbidities and medication, as well as duration of surgery, intra- and postoperative complications were recorded.

### Surgical intervention

**Fig. 2:**
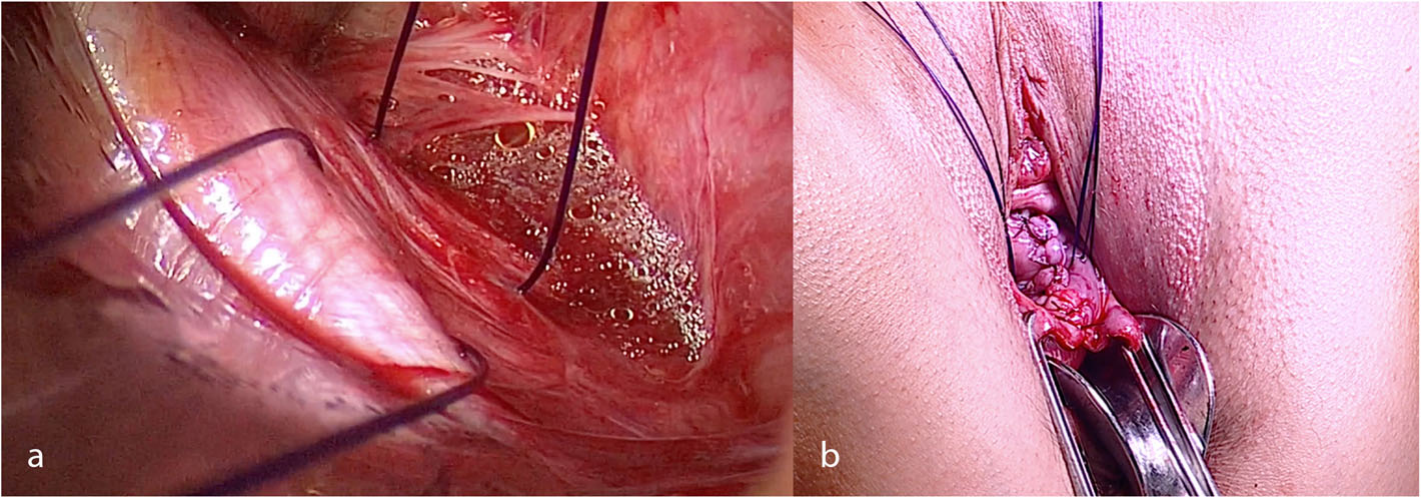
Sacrospinous ligament fixation technique: (**a**) - two PDS 0 (Ethicon, LLC) absorbable sutures were placed in the middle third of the sacrospinous ligament, (**b**) - the sutures passes through the full thickness of the vaginal fornices, the vaginal cuff was closed transversely

All patients had a vaginal hysterectomy, anterior and posterior vaginal wall repair and bilateral sacrospinous ligament fixation of the vaginal cuff. No concomitant incontinence surgery was performed. Vaginal hysterectomy was done in a standard manner. The peritoneal closure was followed by anterior colporrhaphy. After a posterior midline colpotomy a blunt dissection of the pararectal space on both sides was performed. After visualisation of the sacrospinous ligament (SSL) and palpation of the ischial spine, two PDS 0 (Ethicon, LLC) absorbable sutures were placed in the middle third of the SSL, between the sacrum and the ischial spine on each side, without the use of a suture placing device (Fig. 2a). We used three Breisky retractors (at 12, 4 and 8 o’clock axis) to visualise the area and the neddle was passed through the ligament under sight using a long needle holder. No additional lightning was used. Then, the sutures were passed through the full thickness of the vaginal fornices. The vaginal cuff was closed transversely to avoid narrowing the upper vagina and compressing the rectum (Fig. 2b). After a levator ani myorrhaphy was performed, the 4 sutures were tied over the vaginal mucosa so that the vaginal cuff is in contact with the right and left SSL. This was followed by the perineorraphy as last step. All surgeries were performed by the same experienced gynaecologist (CR).

### Statistical analysis

Approximately normally distributed variables were characterized by mean ± standard deviation whereas for other continuous variables median and [minimum to maximum] were given. For the decision on normality, a Shapiro-Wilk test was used. Categorical data were described by numbers and percentages. The changes in POP-Q stages and pelvic floor questionnaire scores between baseline, first follow-up and second follow-up were assessed by the paired Wilcoxon rank test as these variables were considered ordinal respectively were not normally distributed. A significance level of 5% was chosen. All statistical analyses were done using R, version 3.5.1 (R Foundation for Statistical Computing, Vienna, Austria).

## Results

Between March 2016 and March 2017, 21 women with POP stage IV were screened and one was excluded because of other health issues. Twenty women with uterovaginal POP stage IV were included in the study. All of them had a vaginal hysterectomy, an anterior and posterior colporrhaphy and a bilateral SSLF between March 2016 and March 2017. At the time of the surgery, the patients were 68.0 + 6.3 years old. Their mean body mass index (BMI) ranged from 17.7 kg/m^2^ to 34.3 kg/m^2^ with a mean of 24.6 + 4.3 kg/m^2^.

Fourteen out of 20 women had up to two vaginal deliveries, five women had three and one patient had four deliveries. Only one patient smoked, while three women suffered from diabetes mellitus and another one suffered from asthma bronchiale. Median operating time was 114 [90 to 240] minutes, median blood loss was 100 [50 to 150] ml. The postoperative course was uneventful for all patients, and no patient reported excessive buttock pain after surgery. No one required suture removal, while all of them took a course of diclofenac in standard dosage for 5–7 days. No serious adverse events were reported for the duration of the study. Two patients asked for a treatment before the first study follow-up because of an exacerbation of their stress urinary incontinence and received a retropubic tension-free vaginal tape (TVT). The first six-month follow-up was performed after a median of 182.4 [157 to 272] days, the second twelve-month follow-up took place after a median of 366 [342 to 456] days.

Table 1 shows the anatomical outcomes. According to the POP-Q system, the POP stage was significantly improved at six and twelve months after surgery when compared to baseline (*p <* 0.001). All women were asymptomatic at both follow-up visits after surgery and had either a stage 0 or stage I POP, with exception of one woman. The latter presented at the first follow-up visit with a stage III symptomatic cystocele and received an anterior vaginal repair using an anterior mesh.

**Table 1:** Anatomical outcome six and twelve months after vaginal hysterectomy, native tissue cystocele and rectocele repair and bilateral sacrospinous ligament fixation for stage IV pelvic organ prolapse treatment

Number of patients	POP-Q Stage					n
	0	I	II	III	IV	
Baseline	0	0	0	0	20	20
First follow-up (6 months)	10	4	0	1	0	15
Second follow-up (12 months)	11	6	0	0	0	17

Total vaginal length was significantly shorter at first and second follow-up when compared to baseline (median 9 cm vs 7 cm, *p =* 0.008, vs 8 cm, *p =* 0.005). Likewise, the genital hiatus was shorter (median 5 cm vs 2 cm, *p =* 0.001, vs 2 cm, *p <* 0.001) and the perineal body was longer (median 3 cm vs 3 cm, *p =* 0.116, vs 4 cm, *p =* 0.033) (Table 2).

**Table 2:** Vaginal length, genital hiatus and perineal body at baseline, at six and twelve months after vaginal hysterectomy, native tissue cystocele and rectocele repair and bilateral sacrospinous ligament fixation for stage IV pelvic organ prolapse treatment

Median [minimum to maximum]	Vaginal length (cm)	Genital hiatus (cm)	Perineal body (cm)
Baseline	9 [7 to 15]	5 [2 to 9]	3 [2 to 6]
First follow-up (6 months)	7 [6 to 10]	2 [1 to 4]	3 [3 to 5]
Second follow-up (12 months)	8 [6 to 10]	2 [1 to 3.5]	4 [3 to 5]
Difference between Baseline and First follow-up	1 [−1 to 5]	2.5 [0 to 5]	−1 [−2 to 3]
Difference between Baseline and Second follow-up	1 [−1 to 5.5]	2.5 [0.5 to 5.5]	−1 [−2 to 3]
Difference between First and Second follow-up	0 [0 to 1]	0 [−1 to 1]	0 [−1 to 0]

The total score and all four subdomains of the pelvic floor questionnaire showed significant improvement at the first follow-up visit and remained significantly better than baseline at the second follow-up visit (all *p ≤* 0.006), except sexual function (*p =* 0.097 resp. *p =* 0.100).

**Fig. 3:**
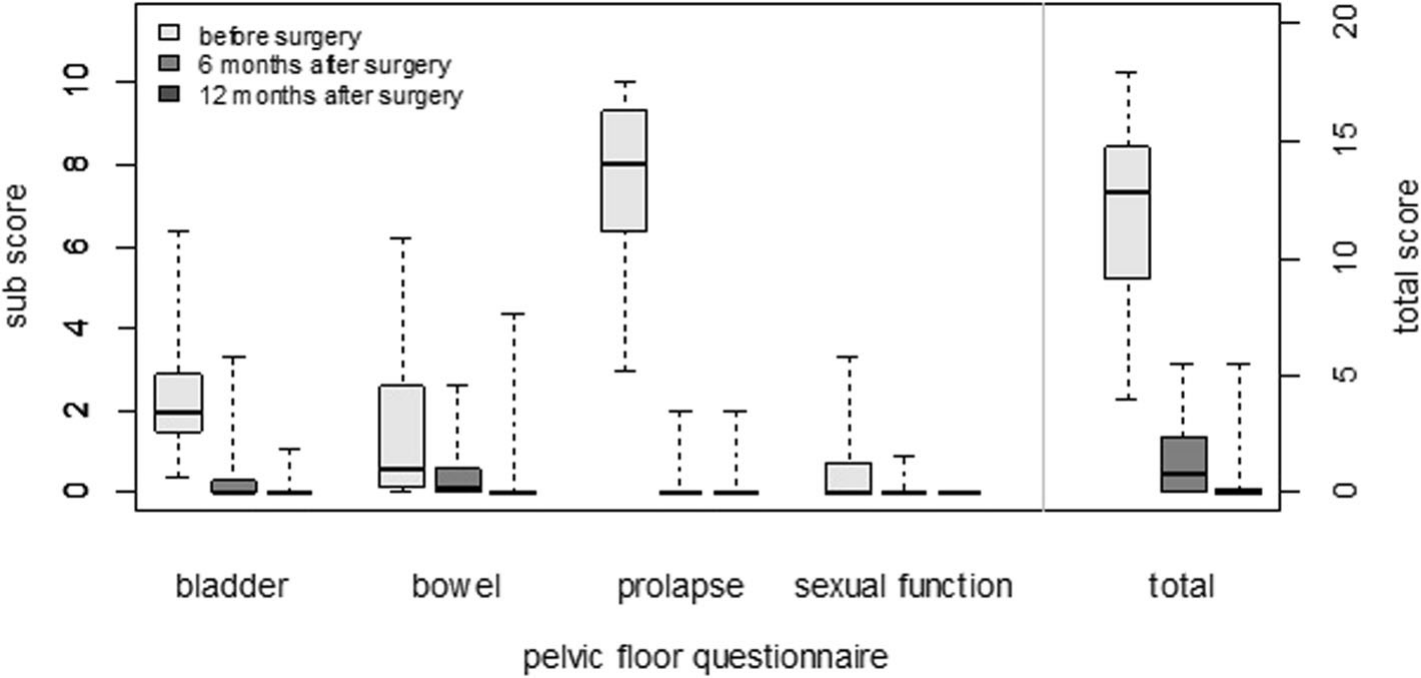
Total scores and subscores of the pelvic floor questionnaire at baseline (before surgery), at the first follow-up (six months after surgery) and at the second follow-up (twelve months after surgery)

Total median scores of the pelvic floor questionnaire were also improved at first follow-up visit when compared to baseline and further improved at the second follow-up (12.8 vs 0.8 vs 0.0) (Fig. 3). Patients who were sexually active before surgery (*n =* 8), continued to be so at the twelve-month follow-up with the exception of one patient due to her husband’s illness. One woman, who was sexually inactive before surgery due to her prolapse, reported a satisfying sexual function after surgery.

All patients were satisfied with the results of the surgical intervention and rated their health condition as “much better” on the Likert scale, both at first and second follow-up visits.

## Discussion

The aim of this prospective case series was to assess the pelvic floor symptoms and anatomical results after vaginal hysterectomy with anterior and posterior vaginal wall repair, combined with bilateral SSLF for the primary treatment of uterovaginal POP stage IV. Scores in all four domains of the pelvic floor symptom questionnaire (bladder, bowel, prolapse, sexual function) were significantly improved at both follow-ups (*p* < 0.001). According to the POP-Q system, the POP stage was significantly improved at six and twelve months after surgery when compared to baseline (*p <* 0.001). All women were asymptomatic at both follow-up visits after surgery and had either a stage 0 or stage I POP, with exception of one woman. The latter presented at the first, six-month follow-up visit with a stage III symptomatic cystocele and received an anterior vaginal repair using an anterior mesh. The patient has had a previous accident with pelvic ring fracture. Whether this might have compromised the stability of the pelvic floor, or the recurrence represents a simple failure of surgery is not clear. The patient was completely asymptomatic after the second, twelve-months follow-up.

The surgical treatment of POP stage IV is challenging. Advanced POP is often combined with vaginal wall ulcerations so that the use of synthetic material becomes a risk factor or completely inappropriate. Furthermore, the excessive lengthening of the vagina may impose difficulties in an abdominal approach as the vaginal cuff may reach the fixation point (e.g. promontorium) and lead to unsatisfactory anatomical results. Abdominal hysterectomy and concomitant sacral colpopexy could lead to further increase of the total vaginal length [11]. Abdominal concomitant total hysterectomy and sacrocolpopexy increases operating time and costs and is associated with more complications [1, 12].

Another important factor in the conceptualisation of this study is the increasing trend of moving away from the use of synthetic graft material for the treatment of primary POP. After the US Food and Drug Administration (FDA) released two public health notifications regarding the potential complications of transvaginal POP repair with synthetic mesh in 2008 and 2011, transvaginal surgical meshes showed a substantial decline. In England, the number of SSLFs grew more than 3 times over the years, whereas sacrocolpopexy remained stable during the considered period of time (2005–2016) [13]. Furthermore, patients’ information through media has increased. According to published data, more than two thirds of patients with POP are informed about their condition and treatment options. In many cases, they reject any treatment, which involves synthetic materials [14, 15]. Hence, there is an emerging need to offer patients with advanced prolapse, who need apical support, efficient treatment options.

Traditionally, SSLF is performed unilaterally, mostly on the right side of the patient. Although it was initially meant as a treatment for post-hysterectomy vaginal vault prolapse, it is now a common procedure performed concomitant to vaginal hysterectomy for uterovaginal prolapse [16]. As treatment of posthysterectomy vault prolapse, Hewson reported a high initial overall satisfaction rate of approximately 90%, and this rate was maintained at 80%, after 4 years [17]. Reports that are more recent show distinctively modest results. Barber et al. in the OPTIMAL randomised trial reported a surgical success rate after SSLF of 60.5%, at 2 years postoperative [18]. Five years postoperative, the estimated surgical failure rate was 70.3% in the SSLF group [19]. The SSLF procedure, performed unilaterally was a modification of the Michigan 4-wall technique.

Arguing for a bilateral and against a unilateral approach, some authors claimed that a bilateral suspension provided a symmetrical and better support of the vaginal vault, while previous concerns of increased morbidity and an abnormal ‘stretching’ of the upper vaginal walls have not been confirmed [20]. However, available data for the bilateral SSLF and specifically for the impact on pelvic floor symptoms is still sparse. Febbraro et al. included 34 patients with POP stage III and IV (according to the American Urogynecologic Society classification, 1996), 24 with and 10 women without uterus, in a feasibility study using a stapler for the fixation of the vaginal cuff on the SSL. After an average of nineteen-month follow-up (range 9 to 32), the authors showed a perfect anatomic result in 77% of cases, one vaginal cuff prolapse recurrence, one cystocele stage II and three patients with vaginal shortening (< 6 cm), while there was one case of a rectal injury through the staple branch, and in 2 patients a small rectal laceration occurred. No postoperative constipation was noted [21]. A more recent report from Mothes et al. in 2015 presents results of 110 women who received a modified, minimal tension, bilateral SSLF [22]. The follow-up was at approximately 14 months and the authors report a 94.5% objective and 96% subjective cure respectively. They also report a 5.5% (6 out of 110 women) apical prolapse and 8.3% (9 out of 110 women) anterior compartment recurrence rate, while only 2/110 and 6/110 patients respectively needed surgical treatment. Despite the very satisfying results at 14 months follow-up, the authors’ approach remains difficult to reproduce due to the mainly subjective suture placement and it does not address the tissue bridging issues and possible insufficient support due to the lack of contact between vaginal wall and ligament tissue. Salvatore et al. in 2018 presented data from a small cohort of patients, who received bilateral SSLF after a second recurrence of prolapse and he showed that at twelve months follow-up, 90% of the patients were cured and reported significant improvement in quality of life and sexual function [23]. The authors used a suturing device and combined absorbable and non-absorbable sutures, while maintaining the classical approach of the SSLF, in contrast to the method of Mothes et al.

Our approach consisted of traditional native tissue repair with absorbable sutures for the SSLF, trying to prevent local foreign body reaction. We also used no suturing device but preferred the visualisation of the ligament for the placement of the sutures, in order to fully control their exact positioning and avoid device related complications such as rectum or nerve injury. As far as the symptoms of the patients in our cohort are concerned, the most important findings, except for the somewhat anticipated improvement of the prolapse scores are the overall recovery in all domains, the great patients’ satisfaction and the consistency of the improvement over the follow-up time. In addition, the anatomical results in our patients were excellent. There was only one symptomatic cystocele five months after the initial treatment, which required surgical intervention with an anterior mesh, while all patients at second follow-up presented with POP-Q Stage either 0 or I.

Obviously, there are limitations to our study. The follow-up time of 12 months is considered short and long-term efficacy could be evaluated. Also, the interpretation of the results should be made with caution, mainly due to the small number of patients included in the analysis, which does not allow extrapolation of the results in larger patient groups. Also, all procedures were performed by the same experienced surgeon, so clustering could be a limiting factor in the interpretation of the results. Furthermore, our patient cohort consisted of women with normal mean BMI, who presented with a primary case of prolapse, without any previous pelvic floor surgery. It remains unknown, if more complex cases, including more risk factors would show the same excellent anatomical and functional results. One should consider, however, that this study was primarily meant to include a homogenous group of patients, in order to be able to objectify the results of a surgical intervention, which has been poorly investigated.

## Conclusion

The results presented in this paper indicate that bilateral SSLF combined with vaginal hysterectomy, native tissue cystocele and rectocele repair is a safe and effective choice for the treatment of an advanced uterovaginal prolapse in women with no previous pelvic floor surgery. Taking into consideration important knowledge from previously published studies, which also showed low morbidity and high satisfaction rates and symptom improvement, this method could be offered as an option to patients, who do not wish the use of alloplastic material in primary prolapse treatment. Nonetheless, long-term effectiveness should be rigorously explored.

## Data Availability

All data was retrieved from the patients’ charts and the source documents. Data is not accessible for the public and permission to access was obtained upon the approval of the local ethics committee.

## Abbreviations

POP: Pelvic organ prolapse
SSLF: Sacrospinous ligament fixation

## References

[1] Haylen BT, Maher CF, Barber MD, Camargo S, Dandolu V (2016). International Urogynecological association (IUGA) / international continence society (ICS) joint report on the terminology for pelvic organ prolapse. Int Urogynecol J.

[2] Slieker-ten Hove M, Pool-Goudzwaard A, Eijkemans M, Steegers-Theunissen R, Burger C, Vierhout ME (2009). Prediction model and prognostic index to estimate clinically relevant pelvic organ prolapse in a general female population. Int Urogynecol J.

[3] Committee on Practice Bulletins - Gynecology, American Urogynecologic Society (2017). Practice bulletin no. 185: pelvic organ prolapse. Obstet Gynecol.

[4] Jha S, Cutner A, Moran P (2018). The UK National Prolapse Survey: 10 years on. Int Urogynecol J.

[5] Shkarupa D, Kubin N, Pisarev A, Zaytseva A, Shapovalova E (2017). The hybrid technique of pelvic organ prolapse treatment: apical sling and subfascial colporrhaphy. Int Urogynecol J.

[6] Barber MD, Maher C (2013). Apical prolapse. Int Urogynecol J.

[7] Rooney K, Kenton K, Mueller ER, FitzGerald MP, Brubaker L (2006). Advanced anterior vaginal wall prolapse is highly correlated with apical prolapse. Am J Obstet Gynecol.

[8] Petri E, Ashok K (2011). Sacrospinous vaginal fixation - current status. Acta Obstet Gynecol Scand.

[9] Baessler K, O'Neill SM, Maher CF, Battistutta D (2010). A validated self-administered female pelvic floor questionnaire. Int Urogynecol J.

[10] Baessler K, Kempkensteffen C (2009). Validation of a comprehensive pelvic floor questionnaire for the hospital, private practice and research. Gynakol Geburtshilfliche Rundsch.

[11] De La Cruz JF, Myers EM, Geller EJ (2014). Vaginal versus robotic hysterectomy and concomitant pelvic support surgery: a comparison of postoperative vaginal length and sexual function. J Minim Invasive Gynecol.

[12] Maher C, Feiner B, Baessler K, Schmid C (2013). Surgical management of pelvic organ prolapse in women. Cochrane Database Syst Rev.

[13] Zacche MM, Mukhopadhyay S, Giarenis I (2018). Trends in prolapse surgery in England. Int Urogynecol J.

[14] Tenggardjaja CF, Moore CK, Vasavada SP, Li J, Goldman HB (2015). Evaluation of patients' perceptions of mesh usage in female pelvic medicine and reconstructive surgery. Urology.

[15] Brown LK, Fenner DE, Berger MB, Delancey JOL, Morgan DM (2013). Defining patients' knowledge and perceptions of vaginal mesh surgery. Female Pelvic Med Reconstr Surg.

[16] Chendrimada KM, Hashim H. Surgery for pelvic organ prolapse. Eur Urol Suppl. 2018:119–25.

[17] Hewson AD (1998). Transvaginal sacrospinous colpopexy for posthysterectomy vault prolapse. Aust NZJ Obstet Gynecol.

[18] Barber MD, Brubaker L, Burgio KL (2014). Comparison of two transvaginal surgical approaches and perioperative behavioral therapy for apical vaginal prolapse: the OPTIMAL randomized trial. JAMA.

[19] Jelovsek JE, Barber MD, Brubaker L, Norton P, Gantz M (2018). Effect of uterosacral ligament suspension vs sacrospinous ligament fixation with or without perioperative behavioral therapy for pelvic organ vaginal prolapse on surgical outcomes and prolapse symptoms at 5 years in the OPTIMAL randomized clinical trial. JAMA.

[20] Shull BL, Capen CV, Riggs MW, Kuehl TJ (1993). Bilateral attachment of the vaginal cuff m 16. Iliococcygeus fascia: an effective method of cuff suspension. Am J Obstet Gynecol.

[21] Febbraro W, Beucher G, Von Theobald P, Hamel P, Barjot P (1997). Feasibility of transvaginal bilateral fixation to the sacrospinous ligament with a stapler: prospective study of the first 34 cases. J Gynecol Obstet Biol Reprod.

[22] Mothes AR, Wanzke L, Radosa MP, Runnebaum IB (2015). Bilateral minimal tension sacrospinous fixation in pelvic organ prolapse: an observational study. Eur J Obstet Gynecol Reprod Biol.

[23] Vitale SG, Laganà AS, Noventa M, Giampaolino P, Zizolfi B, et al. Transvaginal bilateral sacrospinous fixation after second recurrence of vaginal vault prolapse: efficacy and impact on quality of life and sexuality. Biomed Res Int. 2018 Article ID:5727165. 10.1155/2018/5727165 PMC5851336 29675427

